# Development and Mechanical Characterization of Environmentally Friendly PLA/Crop Waste Green Composites

**DOI:** 10.3390/ma18153608

**Published:** 2025-07-31

**Authors:** Karolina Ewelina Mazur, Tomasz Wacław Witko, Alicja Kośmider, Stanisław Tadeusz Kuciel

**Affiliations:** 1 Department of Materials Engineering, Faculty of Materials Engineering and Physics, Cracow University of Technology, Warszawska 24, 31-155 Cracow, Poland; kmazur@uek.krakow.pl (K.E.M.); akosmider12@gmail.com (A.K.); 2 Department of Technology and Ecology of Products, Cracow University of Economics, Rakowicka 27, 31-510 Cracow, Poland; witkot@uek.krakow.pl; 3 Interdisciplinary Centre for Circular Economy, Cracow University of Technology, Warszawska 24, 31-155 Cracow, Poland

**Keywords:** eggs shells, seashells, walnuts, coffee grounds, hydrolytic degradation

## Abstract

This study presents the fabrication and characterization of sustainable polylactic acid (PLA)-based biocomposites reinforced with bio-origin fillers derived from food waste: seashells, eggshells, walnut shells, and spent coffee grounds. All fillers were introduced at 15 wt% into a commercial PLA matrix modified with a compatibilizer to improve interfacial adhesion. Mechanical properties (tensile, flexural, and impact strength), morphological characteristics (via SEM), and hydrolytic aging behavior were evaluated. Among the tested systems, PLA reinforced with seashells (PLA15S) and coffee grounds (PLA15C) demonstrated the most balanced mechanical performance, with PLA15S achieving a tensile strength increase of 72% compared to neat PLA. Notably, PLA15C exhibited the highest stability after 28 days of hydrothermal aging, retaining ~36% of its initial tensile strength, outperforming other systems. In contrast, walnut-shell-filled composites showed the most severe degradation, losing over 98% of their mechanical strength after aging. The results indicate that both the physicochemical nature and morphology of the biofiller play critical roles in determining mechanical reinforcement and degradation resistance. This research underlines the feasibility of valorizing agri-food residues into biodegradable, semi-structural PLA composites for potential use in sustainable packaging or non-load-bearing structural applications.

## 1. Introduction

The growing demand for sustainable materials has intensified research into biodegradable polymers reinforced with natural fillers derived from agricultural and food industry waste [1,2]. Polylactic acid (PLA), a widely used biodegradable thermoplastic, has attracted significant attention due to its renewability, compostability, and suitability for various processing methods, including 3D printing and injection molding. However, its relatively low thermal resistance, brittleness, and sensitivity to environmental degradation significantly limit its application scope, particularly in demanding conditions such as high-temperature environments, mechanically stressed components, or products intended for long-term outdoor use, where material stability, durability, and resistance to aging are critical performance criteria [3,4,5]. In an effort to improve the functional properties of PLA while enhancing its environmental credentials, recent studies have focused on the incorporation of organic fillers that not only originate from renewable resources but also contribute to waste valorization [6,7,8]. The global production of food waste is a significant environmental concern, with substantial volumes of organic waste generated annually. Among the most commonly discarded food by-products are coffee grounds, eggshells, walnut shells, and seashells. In 2020, it was estimated that over 9 million tons of coffee grounds were discarded worldwide, contributing to landfill waste despite their rich organic content and potential for reuse [9]. Similarly, eggshells, as a by-product of the egg industry, are produced in vast quantities, with approximately 7.2 million tons generated globally each year [10]. Walnut shells, generated during the processing of walnuts, account for around 2.6 million tons annually and are typically disposed of as waste [11,12]. Seashells, predominantly from shrimp, lobster, and crab, are produced in massive amounts, between 6 and 8 million tons globally each year [13,14].

In this study, PLA-based composites were developed using four types of bio-based waste materials: coffee grounds, eggshells, walnut shells, and seashells. These additives were selected due to their availability, compositional diversity (e.g., calcium carbonate, lignocellulosic content), potential influence on the mechanical and degradation behavior of the composite materials, and their abundant availability, low cost, and potential to enhance material properties. Coffee grounds, rich in organic compounds and fibers, contribute to the improvement of mechanical strength and reduce the composite’s overall density [15,16,17]. Essabir et al. demonstrated in their study that the incorporation of coffee grounds as a filler in polyethylene (PE) composites led to a significant increase in the Young’s modulus of the material. Specifically, a 5 wt% addition of coffee grounds resulted in a 20% increase in the modulus, while a 20 wt% filler content enhanced it by up to 50% [18]. Eggshells, primarily composed of calcium carbonate, enhance the stiffness and thermal stability of PLA, while also providing bioactive properties [19,20]. Orisekeh et al. [21] demonstrated that the incorporation of eggshell particles into PLA did not significantly influence the tensile or flexural strength of the composite; however, it led to an improvement in its compressive properties, with the sample containing 5 wt% eggshells achieving an average compressive strength of 66.50 MPa and a modulus of 2.27 GPa. Additionally the addition of eggshell particles did not enhance the thermal properties of PLA; in fact, the neat PLA exhibited higher glass transition, crystallization, and melting temperatures measured at 62 °C, 127 °C, and 151 °C, respectively. In Boronat’s work [22], an increasing proportion of eggshells in the PE-based composite formulation led to a linear decrease in enthalpy and a delay in the onset of thermal degradation, while the melting temperature remained unaffected. Walnut shells, known for their hardness and fibrous nature, offer significant improvements in impact resistance and surface texture [23,24], whereas seashells, rich in chitin and chitosan, further increase the composite’s strength and add antimicrobial properties [23,25,26]. In the work by Moustafa et al., the mechanical and tribological properties of polypropylene (PP) and walnut shell powder were investigated at three filler weight fractions: 30%, 40%, and 50%. The results indicated that increasing the walnut shell powder content by 20 wt% led to a reduction in ultimate tensile and bending stress by 19.5%, as well as a 7.7% decrease in the modulus of elasticity. Conversely, this reduction in strength and stiffness was accompanied by a 5% increase in surface hardness. Additionally, improvements were observed in impact resistance and elongation at break [27]. Prasad et al., in their study on the reinforcing effect of powdered seashells in epoxy resins, demonstrated that the most favorable results were obtained at a 5% filler content. At this concentration, the Vickers hardness of the material increased from 21 to 24.5, while the ultimate tensile strength rose significantly from 119.2 MPa to 246.6 MPa. Additionally, the flexural strength improved from 246 MPa to 344.9 MPa, representing an increase of nearly 68% [28]. As shown in previous studies, the incorporation of these natural additives into PLA composites not only can improve their mechanical, thermal, and antimicrobial performance but also aligns with the principles of sustainable development by utilizing waste materials. Additionally, the properties of these composites can be further optimized by adjusting the size, morphology, and loading content of the fillers, as well as exploring surface modification techniques to improve the interfacial bonding between PLA and additives.

Research on PLA enriched with natural additives is innovative due to its focus on sustainability, circularity, and the development of bio-based composites with improved functional properties. While extensive studies have already been conducted on incorporating similar organic fillers into traditional petroleum-based polymers like PP [29,30,31], polyethylene (PE) [32,33], and polyvinyl chloride (PVC) [34,35,36], these efforts largely aimed at cost reduction or mechanical reinforcement without significantly addressing biodegradability or life cycle performance [37,38]. In contrast, the integration of natural waste materials into PLA not only opens new pathways for valorizing food and agricultural by-products but also supports the transition toward fully compostable and environmentally friendly materials particularly important in packaging, biomedical, and consumer product applications.

Issues related to fiber treatment and water absorption play a critical role in the design of bio- and green composites. Despite their low density, renewability, and favorable strength-to-weight ratio, natural fibers exhibit high hygroscopicity, which can lead to the deterioration of mechanical properties and the reduced durability of the composite under elevated humidity conditions. Proper surface treatment of the fibers, such as chemical modifications including alkalization, silanization, or acetylation, can enhance their compatibility with the polymer matrix, reduce water uptake, and improve interfacial adhesion. These measures contribute to more uniform stress transfer and better long-term performance of the composite. As demonstrated in the literature [39], both effective fiber treatment and moisture control are essential to achieving stable and predictable properties in bio-based composites. Neglecting these factors may result in an incomplete assessment of the material’s functional behavior and its performance under real-world conditions. Moreover, moisture significantly affects mechanical parameters and the degradation of the fiber–matrix interface, which is especially important for applications exposed to variable environmental conditions. Therefore, a comprehensive analysis of these aspects should be an integral part of both the design and characterization of biocomposites. Including such considerations is essential for accurately evaluating the application potential of materials derived from renewable resources.

The prepared PLA-based composites were subjected to comprehensive mechanical characterization including tensile strength and Charpy impact resistance tests to evaluate the reinforcing effect of various waste-derived fillers. Furthermore, to simulate real-world service conditions, hydrolytic aging was performed to assess moisture-induced degradation. Unlike earlier studies that typically focus on a single filler type or neglect environmental durability, this work systematically compares multiple agro-industrial residues (e.g., spent coffee grounds, walnut shell powder, crustacean and eggshell biowastes) processed by injection molding. By correlating filler morphology, interfacial adhesion, and water uptake kinetics with long-term mechanical retention, we identify promising waste-derived additives that not only enhance initial performance but also maintain durability under hydrolytic stress. To our knowledge, this is the first study to integrate standardized hydrolytic degradation testing with both tensile and impact property evaluation across a broad suite of PLA biocomposites, thereby offering a truly holistic assessment of eco-friendly, durable materials for additive manufacturing.

## 2. Materials and Methods

### 2.1. Material and Composite Preparation

PLA (Type: PLI 005) supplied by NaturePlast (Ifs, France) was used as the base material. PLI 005 is a material produced from non-genetically modified, renewable plant resources. It is recyclable, biodegradable, and compostable industrial conditions. It has the form of transparent granules and is intended for injection molding, including injection blow molding. Table 1 presents the basic values of the parameters characterizing this material.

#### 2.1.1. Additive Processing

In order to valorize food waste, the following fillers were selected as modifying additives: seashells (S), eggshells (E), walnut shells (W), and coffee grounds (C). In order to use food waste for the production of composites, they were appropriately prepared. The first stage was their mechanical cleaning of unwanted residues and contaminants. Then, S, E, and W were ground using a universal Retsch ZM 200 grinder (Haan, Germany) with a mesh size of 500 µm. On the other hand, C, due to their high fat content, were subjected to a degreasing process. For this purpose, they were soaked for an hour in a 10% solution of lye (NaOH) with water and then rinsed several times in water. The last step in the preparation of fillers was their drying in a vacuum dryer for 4 h at a temperature of 160 °C and a pressure of 620 mbar.

#### 2.1.2. Composites Processing

Based on the relevant literature data and preliminary experimental findings, the filler content in the composites was set at 15 wt% for each type of natural fiber. Based on our own experience [42] and the work of other researchers [42,43], it can be concluded that the addition of 15 wt% is a compromise between the improvement of Young’s modulus and the decrease in tensile strength. Tensile strength decreases with increasing natural fiber content mainly due to poor adhesion between the matrix and fibers, causing stress concentrations that accelerate sample failure. Additionally, the brittle nature of the fibers reduces the composite’s deformability, limiting its elongation at break.

Prior to processing, PLA was dried for 4 h at 60 °C to minimize moisture-induced degradation during melt processing. To improve the interfacial compatibility between the PLA matrix and the natural fillers, a dedicated compatibilizer (SCONA TPPL 5112 PA, BYK, Wesel, Germany) was incorporated into the composites.

**Figure 1:**
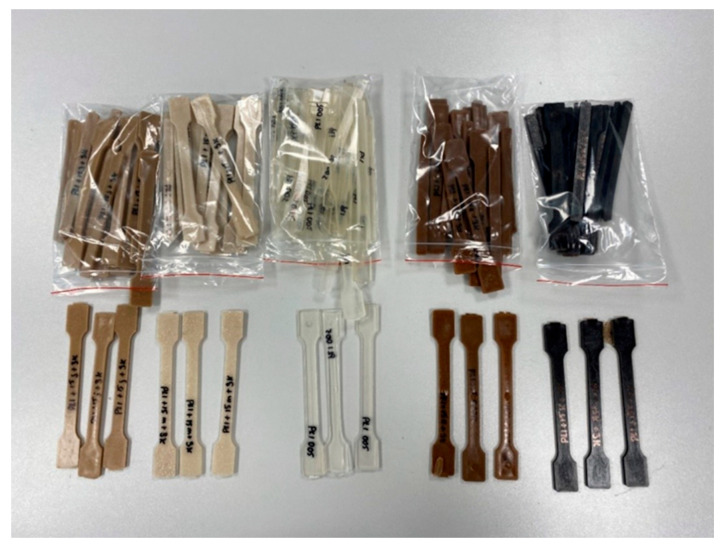
Images of the dumbbell samples of manufactured PLA and its composites.

The composites with varying wt% of fillers are summarized in Table 2 and visualized in Figure 1. Standard dumbbell-shaped specimens were produced using a universal injection molding machine (KM 40-125 Winner, Krauss Maffei, Munich, Germany) in accordance with the PN-EN ISO 3167 [44] standard, with final specimen dimensions of 10 × 4 × 150 mm.

The temperature profile along the injection machine was set as follows: 180 °C (zone 1), 185 °C (zone 2), 190 °C (zone 3), and 190 °C (zone 4), with the nozzle temperature set at 195 °C and the mold temperature maintained at 20 °C. The temperature in the material feed zone was 25 °C. The injection pressure was maintained at 1400 bar throughout the molding process.

### 2.2. Testing Methods

#### 2.2.1. SEM

In order to evaluate the adhesion of the fillers to the polymer matrix as well as the dispersion of the fillers within the matrix, scanning electron microscopy (SEM) imaging was performed. The images were acquired on fracture surfaces obtained after tensile strength testing. Prior to imaging, the sample surfaces were coated with a thin layer of gold using a sputter coater (DII-29030SCTR, JEOL, Tokyo, Japan) to enhance surface conductivity. SEM images were taken using a JEOL JSM-IT200 microscope (Tokyo, Japan) under low vacuum conditions at an accelerating voltage of 15 kV.

#### 2.2.2. Mechanical Tests

One of the fundamental methods for evaluating mechanical strength is the static tensile test. The test was performed using a Shimadzu AGS-X (Kyoto, Japan) universal testing machine (desktop variant) equipped with a 1 kN load cell. The measurement procedure was carried out in accordance with the ISO 527 standard [40]. Three series of tests were conducted for each of the five fabricated types of specimens. The tests were performed at a crosshead speed of 2 mm/min. An extensometer was used to ensure more accurate strain measurements.

The three-point flexural test (ISO178 [45]) was assessed by using an MTS Criterion 43 (Eden Prairie, MN, USA) universal testing machine with the MTS software TestSuites 1.0. The crosshead speed for flexural tests was 10 mm/min with the load cell 30 kN and span length of 80 mm.

The Charpy impact test was conducted in accordance with ISO 179 [41] using a Zwick/Roell MTS-SP testing machine (Ulm, Germany). An impact energy of 5 J was applied to unnotched specimens. The dimensions of the test samples were 4 × 10 mm. This test evaluates the impact resistance of materials based on the absorbed energy during fracture.

#### 2.2.3. Hydrolytic Degradation

In order to evaluate the influence of environmental conditions, hydrolytic degradation of the fabricated composites was performed. The mass change observed during degradation provided insight into the water absorption behavior of the materials and whether the specified exposure time contributed to composite degradation. Water absorption testing was conducted over a period of 28 days at a temperature of 50 °C in a solution of water and an enzymatic dishwashing agent. The mass variation was recorded at time intervals of 1, 7, 21, and 28 days using an Ohaus Adventurer analytical balance (Parsippany, NJ, USA). Subsequently, water absorption was calculated using Equation (1):

(1) $$M_{t}=\left[\frac{W_{t}-W_{0\;}}{W_{0\;}}\right]\times 100,$$

where

*M_t_*—percentage of water content;
*W_t_*—instantaneous weight of the sample;
*W*_0_—the initial weight of the sample.

After four weeks, the samples were removed from the solution and subsequently subjected to tests determining selected properties of the fabricated materials, such as tensile and flexural strength. Optical microscopy images of the samples were taken using a Keyence VHX-7000 microscope (Osaka, Japan) after the first and fourth week of degradation.

To quantitatively describe the phenomenon of water absorption by the material, Fick’s second law was applied. This law describes an unsteady-state diffusion process, in which the concentration of a substance (in this case, water) changes over time and space. This approach is particularly suitable for the initial and intermediate stages of moisture uptake, when the moisture content within different layers of the composite has not yet reached equilibrium and changes dynamically as a function of time. To calculate the diffusion kinetics, the following formula was used:

(2) $$log\frac{M_{t}}{M_{m}}=\log\left(k\right)+\;n\;log(t),$$

where

*M_m_*—equilibrium water content;

*k* and *n*—constants derived from the slope of the curve and the intercept of the *log(Mt/M∞)* and *log(t)* plots that can be derived from the experimental data.

The diffusion coefficient *D* was calculated using the following formula:

(3) $$\frac{M_{t}}{M_{m}}=\frac{4}{h}{\left(D/\pi \right)}^{1/2}\times t^{1/2},$$

After transforming Equation (3), we can calculate the diffusion coefficient *D* using Equation (4):

(4) $$D=\frac{\pi }{{\left(4M_{m}\right)}^{2}}\times \;\frac{{\left(M_{t}\times L\right)}^{2}}{\sqrt{t}}=\frac{\pi k^{2}}{{\left(4M_{m}\right)}^{2}},$$

All mechanical tests were performed at least 5 times.

## 3. Results

### 3.1. SEM

**Figure 2:**
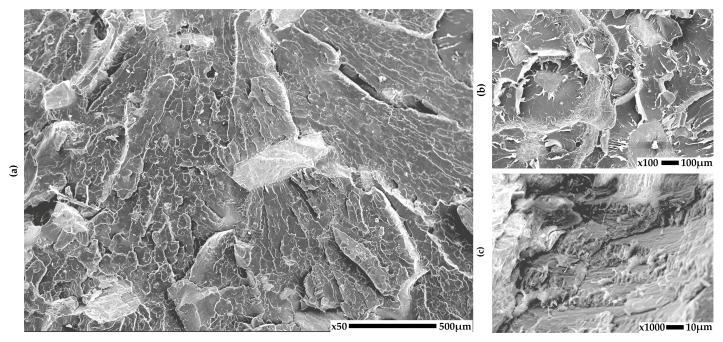
SEM images of PLA composites with seashells: (**a**) 50× (**b**) 100×, (**c**) 1000× magnification.

**Figure 3:**
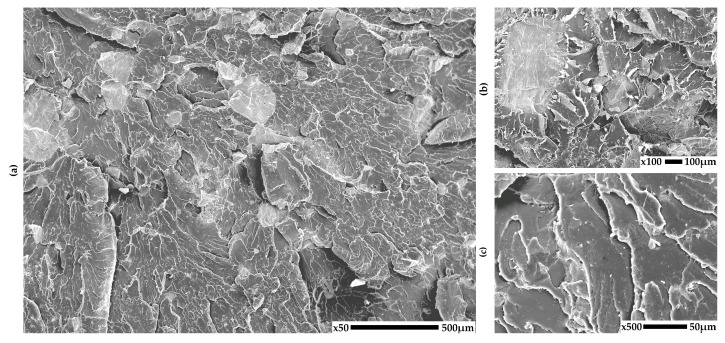
SEM images of PLA composites with eggs shells: (**a**) 50×, (**b**) 100×, (**c**) 500× magnification.

**Figure 4:**
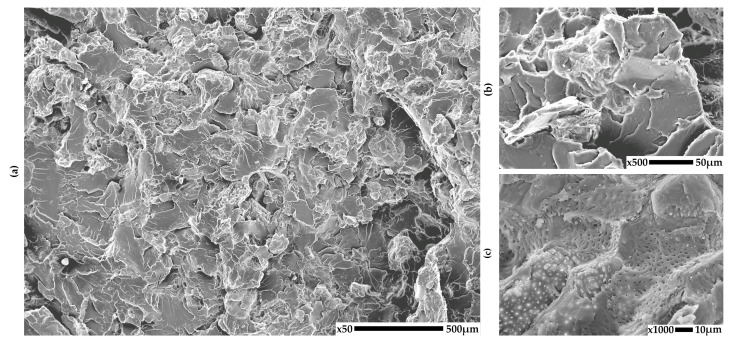
SEM images of PLA composites with walnut shells: (**a**) 50×, (**b**) 500×, (**c**) 1000× magnification.

**Figure 5:**
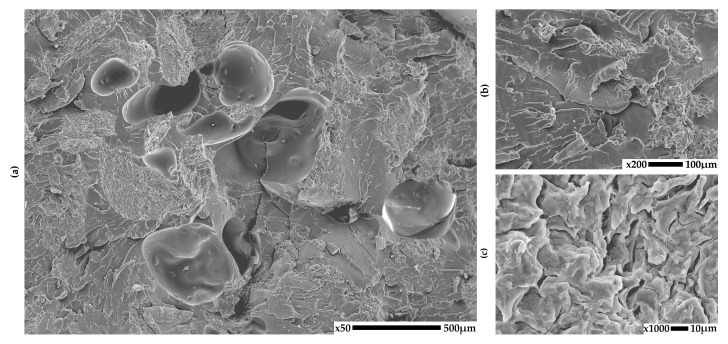
SEM images of PLA composites with coffee grounds: (**a**) 50×, (**b**) 200×, (**c**) 1000× magnification.

The structure of the obtained PLA-based composites was analyzed using scanning electron microscopy (SEM) in order to evaluate the distribution and adhesion of the natural fillers seashells, eggshells, walnut shell powder, and coffee grounds within the polymer matrix. Representative SEM micrographs taken at different magnifications are shown in Figure 2, Figure 3, Figure 4 and Figure 5 for each composite to illustrate the morphology of the fractured surfaces after tensile failure.

The images reveal notable differences in particle dispersion, interfacial bonding, and fracture behavior depending on the type of used filler. For the PLA15S composite (Figure 2), the particles exhibit irregular and angular shapes and are partially embedded in the matrix. Some interfacial voids are visible, suggesting limited adhesion between the hard mineral fillers and the PLA phase. The fractured surface appears relatively flat and layered, which is indicative of brittle fracture dominated by the matrix. Estimated particle size is 20–50 µm.

In contrast, the PLA15E composite (Figure 3) shows a more porous structure with numerous rounder filler particles. The adhesion between the eggshell powder and PLA appears weaker, as evidenced by the occurrence of matrix detachment and the presence of microvoids around the particles. This suggests a less efficient load transfer and a higher likelihood of crack initiation at the filler–matrix interface.

The PLA15W composite (Figure 4) demonstrates the most irregular and rough fracture surface among the tested materials. The walnut shell particles are larger and more porous and appear well anchored in the matrix. This may be attributed to the increased surface roughness of the filler, promoting mechanical interlocking. Despite the presence of voids, the general cohesion between the filler and matrix seems improved compared to the other systems, possibly due to the higher compatibility between the lignocellulosic walnut powder and the PLA matrix.

The PLA15C composite (Figure 5) exhibits a relatively heterogeneous and moderately rough fracture surface compared to the other tested composites. The coffee particles are irregular in shape, with a broad size distribution, and appear only partially embedded within the PLA matrix. In several regions, visible interfacial gaps and particle pull-out suggest limited adhesion between the hydrophilic coffee filler and the hydrophobic polymer matrix (compared by mechanical tests).

Despite these debonding features, localized areas show signs of plastic deformation in the PLA matrix surrounding the particles, which may indicate partial mechanical interlocking. The surface of the coffee particles appears smoother than that of other lignocellulosic fillers, which may contribute to the weaker interaction observed. Overall, the interfacial bonding in PLA15C seems less effective than in the PLA15W system, likely due to lower surface roughness and reduced compatibility between the spent coffee grounds and PLA.

### 3.2. Mechanical Properties

The incorporation of various bio-originating fillers into the PLA matrix significantly influenced the mechanical performance of the resulting biocomposites. Table 3 presents a comparative analysis of tensile strength and Young’s modulus and Figure S1 representative curves for neat PLA and its composites reinforced with seashells (S), eggshells (E), walnut shell powder (W), and coffee grounds (C).

The unmodified PLA exhibited a baseline tensile strength of approximately 32 MPa, consistent with values reported in the literature for injection-molded or 3D-printed PLA-based materials [46,47]. Upon the addition of biofillers, a pronounced increase in tensile strength was observed across all systems, with values ranging from ~45 MPa (PLA15E) to ~55 MPa (PLA). 

Among the studied composites, the PLA15S composite showed the highest tensile strength (~55 MPa), representing a ~72% enhancement over neat PLA. This notable improvement may be attributed to the high calcium carbonate content and the irregular, angular morphology of powdered seashells, which likely promoted effective stress transfer at the matrix–filler interface. Similarly, PLA15C achieved a tensile strength of ~54 MPa, suggesting that coffee grounds also play a reinforcing role, potentially due to their fibrous microstructure and lignocellulosic composition enhancing interfacial adhesion.

PLA15W and PLA15E composites also demonstrated elevated tensile strengths, albeit to a lesser extent (~49 MPa and ~45 MPa, respectively). The moderate enhancement for E/PLA may be due to the relatively fine and uniform particle distribution of eggshell powder but potentially limited interfacial bonding due to the smoother surface morphology and lower aspect ratio. In the case of PLA15W, although the walnut shell filler has high rigidity, the organic and carbonaceous nature of the particles may have led to stress concentrations or weak bonding regions, limiting the improvement.

In contrast to tensile strength, the introduction of biofillers generally resulted in a reduction in Young’s modulus compared to neat PLA, which exhibited a modulus of ~5200 MPa. The most significant decrease was observed for PLA15W, with a modulus of ~4200 MPa, representing a ~20% reduction. The reduction in stiffness is likely associated with poor dispersion or limited interfacial compatibility, leading to microvoids or regions of stress shielding within the matrix.

Similarly, PLA15S and PLA15E composites showed lower modulus values (~4300 MPa and ~4400 MPa, respectively). Although these fillers enhanced strength, they appeared to soften the composite overall, possibly due to the particulate nature of the reinforcements. Interestingly, PLA15C demonstrated the highest modulus among the modified composites (~4800 MPa), indicating that coffee-derived lignocellulosic particles may provide some reinforcing rigidity due to their fibrous structure and relatively high stiffness.

The divergent trends in tensile strength and stiffness suggest that biofillers contributed more significantly to energy dissipation and crack deflection mechanisms, rather than directly increasing composite rigidity. The enhancement in strength, coupled with modulus reduction, points to toughening effects introduced by the fillers—particularly in the PLA15S and PLA15C systems—through mechanisms such as microcrack bridging, stress transfer, and particle–matrix interlocking.

Moreover, the distinct behavior among the fillers underscores the importance of filler morphology, particle size, chemical composition, and surface properties. Calcium-rich fillers (S, E) tend to promote better strength but lower stiffness, whereas lignocellulosic fillers (W, C) show more variability depending on interfacial bonding and particle rigidity.

The mechanical properties of PLA-based composites modified with various natural fillers seashells (S), eggshells (E), walnut shells (W), and coffee grounds (C) were analyzed to evaluate the influence of these bio-based reinforcements on flexural strength, stiffness, and impact resistance (Table 4). Moreover, a representative curves of flexural testes were presented in Figure S2.

The addition of seashells (PLA15S) resulted in a noticeable decrease in flexural strength from 108 ± 0.7 MPa (neat PLA) to 92.6 ± 0.35 MPa, representing a reduction of approximately 14.3%. Simultaneously, the flexural modulus increased by 7.7% to 3742 ± 68 MPa, indicating improved stiffness. However, the impact strength dropped significantly by 56.6%, from 17.94 ± 0.71 to 7.79 ± 0.35 kJ/m^2^. This behavior suggests that the rigid, brittle nature of the mineral filler increased the material’s stiffness but compromised its toughness, likely due to poor interfacial adhesion and stress concentration.

Similarly, the incorporation of eggshells (PLA15E) caused the flexural strength to decline by 17.3% to 89.3 ± 5.75 MPa, while the flexural modulus increased to 3939 ± 219 MPa—a 13.4% improvement. The impact resistance dropped to 8.75 ± 0.40 kJ/m^2^ (a 51.2% reduction). These results are consistent with the behavior of hard particulate fillers, which enhance stiffness at the expense of ductility and impact resistance.

The walnut-shell-filled composite (PLA15W) showed a flexural strength of 85.4 ± 5.95 MPa (20.9% lower than neat PLA) and a modest increase in flexural modulus to 3641 ± 206 MPa (4.8%). Interestingly, PLA15W retained relatively better impact strength (10.65 ± 0.51 kJ/m^2^), showing a smaller decline of 40.6%. This may be attributed to the fibrous structure of the walnut shells, which possibly promotes more effective stress transfer and energy dissipation, making PLA15W the most balanced composite in terms of mechanical performance.

In contrast, the coffee ground composite (PLA15C) exhibited the most significant deterioration in mechanical strength, with flexural strength decreasing by 45.1% to 59.3 ± 0.35 MPa. Nevertheless, this composite demonstrated the highest flexural modulus (4330 ± 53 MPa), a 24.7% increase, indicating strong stiffening capability. However, the impact strength dropped drastically to 6.08 ± 0.27 kJ/m^2^, a 66.1% reduction. This suggests that the porous and possibly agglomerated nature of coffee grounds introduces critical flaws that initiate early failure, severely impairing toughness.

In summary, the introduction of bio-based fillers into PLA led to increased stiffness in all cases, but at the cost of reduced flexural strength and impact resistance. Among the tested fillers, walnut shells offered the most favorable balance between stiffness and toughness, making PLA15W a promising candidate for semi-structural applications. In contrast, coffee grounds, while significantly enhancing stiffness, detrimentally affected overall mechanical integrity, highlighting the need for better filler dispersion or surface treatment strategies. These insights are valuable for designing sustainable, biodegradable composites for 3D printing applications where mechanical performance must be tailored to specific functional requirements.

### 3.3. Hydrolytic Degradation

**Figure 6:**
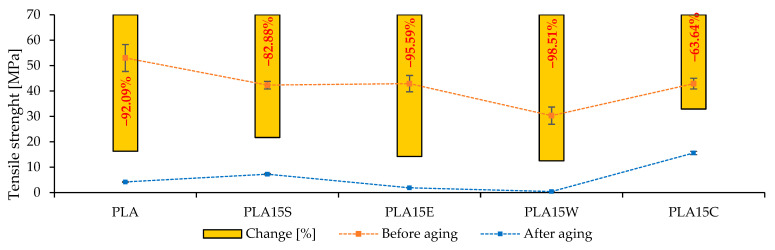
Tensile strength before and after aging of PLA and its composites.

**Figure 7:**
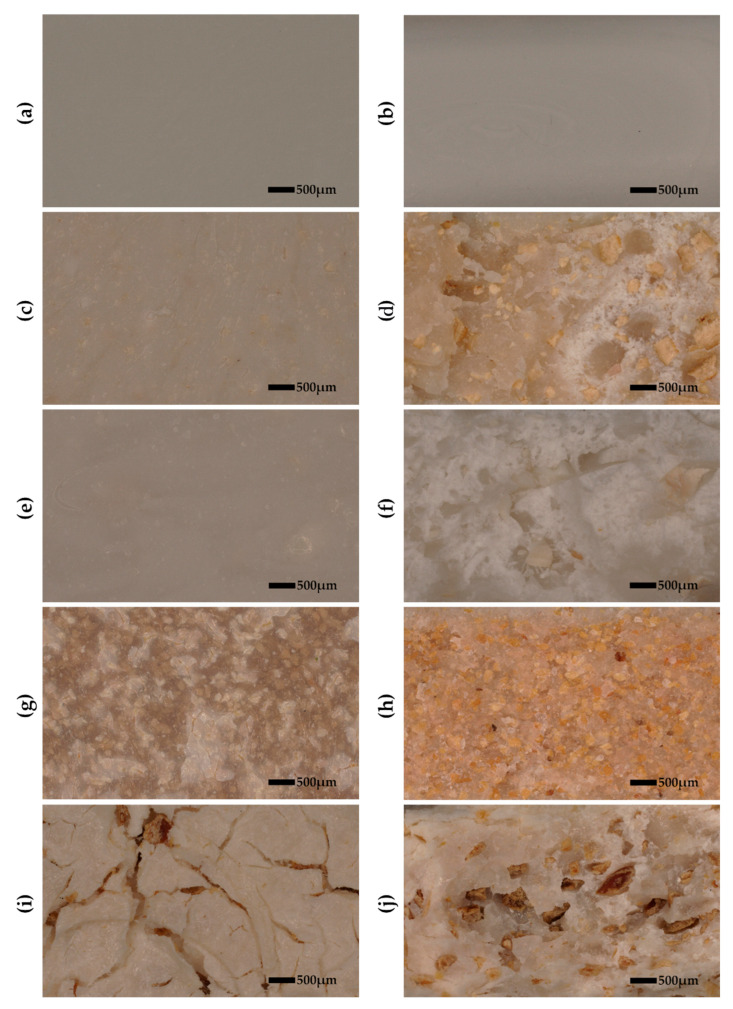
Optical images of breakouts of PLA and its composites before (**left panel**) and after aging (**right panel**): (**a**,**b**)—PLA, (**c**,**d**)—PLA15S, (**e**,**f**)—PLA15E, (**g**,**h**)—PLA15W, (**i**,**j**)—PLA15C.

**Figure 8:**
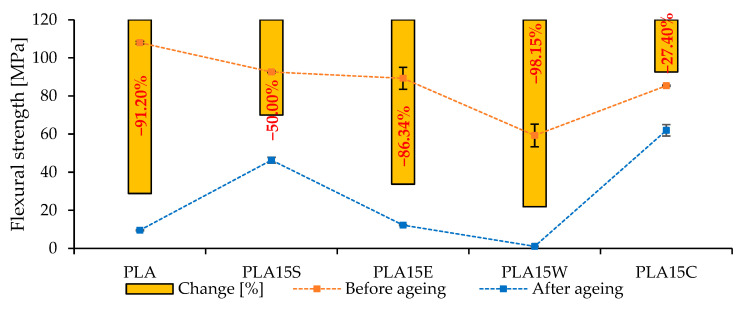
Flexural strength before and after aging of PLA and its composites.

Figure 6 and Figure 8 and Table 5 show the mechanical results after hydroaging, and Figure 7 displays the optical images of breakouts of samples before (left panel) and after (right panel) aging.

The incorporation of seashells into PLA (PLA15S) results in an initial tensile strength of approximately 43 MPa, which decreases to around 7.3 MPa after aging—a reduction of 82.88%. Although this represents a significant decline, it is lower than the degradation observed in neat PLA, suggesting that seashells may contribute to improved durability under hydrothermal aging conditions compared to unmodified PLA and other additives, except for coffee grounds.

In the case of PLA reinforced with 15% eggshells (PLA15E), the tensile strength drops from approximately 43 MPa to just 1.9 MPa after aging, corresponding to a 95.59% decrease. This pronounced degradation likely stems from the high porosity and poor interfacial compatibility between the mineral–organic filler and the PLA matrix, which accelerates structural breakdown under moist conditions.

The PLA composite with walnut shells (PLA15W) exhibits the most severe decline in mechanical performance. The tensile strength falls from about 30 MPa to just 0.45 MPa after aging, indicating a 98.5% reduction. This extreme deterioration is probably due to the high hygroscopicity of walnut shells and their weak interfacial adhesion with the polymer matrix, which promotes moisture uptake and microcrack formation.

Conversely, the PLA composite containing coffee grounds (PLA15C) demonstrates the highest mechanical stability after aging. Although the strength decreases from roughly 44 MPa to 16 MPa (a 63.64% reduction), it still retains the highest post-aging tensile strength among all tested materials. This suggests a favorable dispersion of coffee residues in the PLA matrix, potentially attributed to the stable lignocellulosic nature of coffee particles and their reduced susceptibility to hydrolytic degradation.

In summary, PLA15C appears to be the most promising formulation for applications exposed to hydrothermal environments, due to its relatively high retention of mechanical properties after aging. PLA15S shows moderate resistance, while PLA15E and especially PLA15W exhibit substantial mechanical deterioration.

The flexural strength of PLA-based composites reinforced with various natural fillers was assessed before and after accelerated aging to evaluate their mechanical stability over time (Figure 8). The incorporation of seashells (PLA15S) resulted in a moderate initial flexural strength of approximately 90 MPa, which decreased by 50.00% after aging. This represents a substantial improvement in aging resistance compared to neat PLA, which exhibited a drastic 91.20% loss in flexural strength. Similarly, the addition of eggshells (PLA15E) provided limited benefit; the material retained only 13.66% of its original strength after aging, corresponding to a reduction of 86.34%. The use of walnut shell filler (PLA15W), however, significantly worsened aging performance. The flexural strength dropped by 98.15%, indicating a near-complete degradation of mechanical integrity, likely due to poor interfacial bonding or incompatibility between the lignocellulosic filler and the PLA matrix. In contrast, the coffee-filled composite (PLA15C) demonstrated the most promising performance, with a relatively high initial strength (~75 MPa) and the smallest reduction in mechanical properties (−27.40%). This enhanced stability may be attributed to the antioxidant compounds naturally present in spent coffee grounds, which could inhibit oxidative degradation during aging. Overall, coffee grounds emerged as the most effective filler for improving the long-term flexural performance of PLA composites, followed by seashells, eggshells, and finally walnut shells, which proved detrimental.

**Figure 9:**
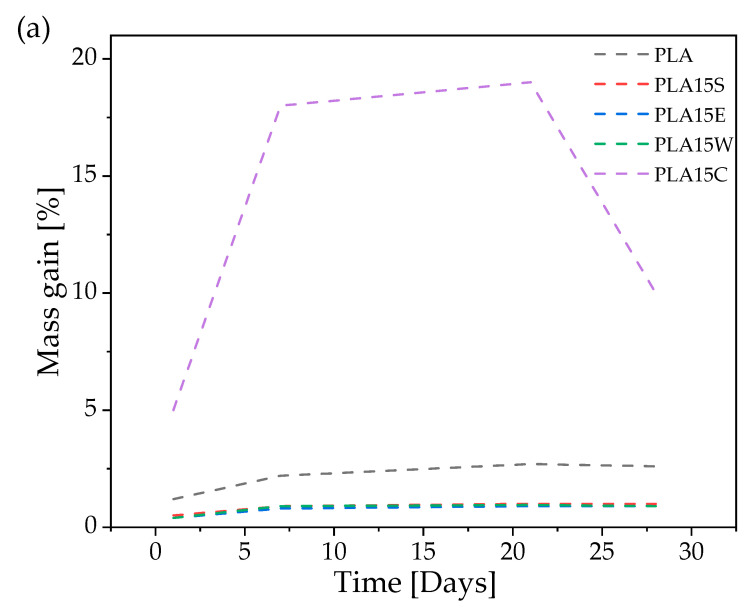

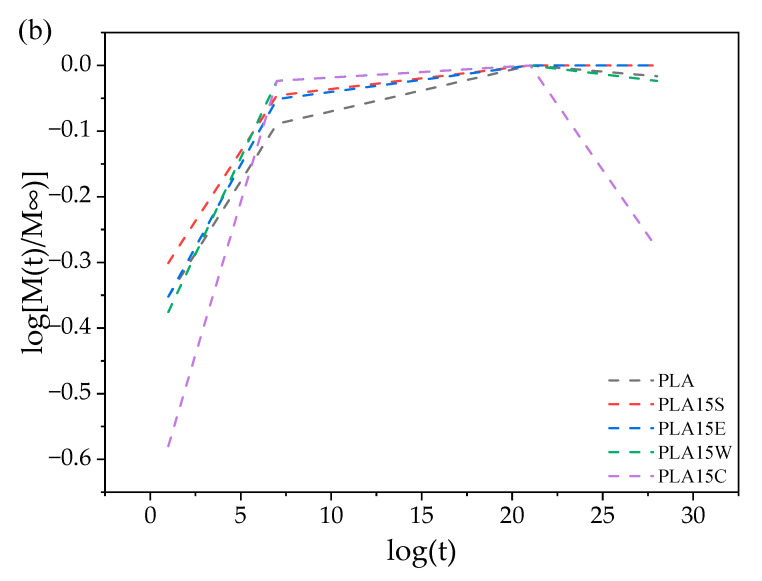
Mass gain (**a**) and kinetics of degradation (**b**) of PLA and its composites.

Figure 9 presents the water uptake behavior (a) and hydrolytic degradation kinetics (b) of neat PLA and its composites filled with various organic waste materials during 30 days of incubation in physiological saline solution, which are compared in Table 6. The results reveal clear differences in water absorption capacity and degradation behavior depending on the type of filler used. Although gravimetric uptake methods offer a simple and rapid means of estimating diffusion coefficients, they are inherently coarse and subject to larger uncertainties compared to sorption or permeability techniques (e.g., dynamic vapor sorption methods and water vapor transmission rate measurements), which provide more accurate and direct quantification of mass transport [48].

The most pronounced water absorption was observed for the PLA15C composite, which contains ground coffee waste. This filler is highly porous and rich in hydrophilic functional groups (–OH, –COOH), which promote rapid water uptake. A sharp increase in mass (~18%) occurred within the first 10 days, reaching over 19% by day 20. This indicates not only high water affinity but also the likely formation of microchannels at the filler/matrix interface, facilitating moisture penetration. The diffusion coefficient (D = 2.51808·10^−12^ m^2^/s) is the lowest among the tested materials, with a low R^2^ value (0.492) and high diffusional exponent (n = 0.2896).

In contrast, the PLA15S composite, containing shell powder derived from seashells (rich in chitin and chitosan), also showed increased water uptake (~2–3%), consistent with the hydrophilic nature of polysaccharides. However, the kinetic parameters (n = 0.2128, R^2^ = 0.933) indicate a more predictable diffusion process, likely governed by Fickian transport.

The PLA15E composite, filled with eggshell powder (primarily proteinaceous), exhibited moderate water uptake. Although egg-derived proteins such as albumins can bind water, the filler structure appears less porous than ground coffee, resulting in a diffusion coefficient (D = 7.1824·10^−12^ m^2^/s) comparable to that of neat PLA, implying a neutral effect on water permeability.

The lowest water absorption was observed in PLA15W, which contains walnut shell powder. This lignocellulosic filler has relatively low polarity and a fatty-acid-rich surface that limits water interaction. The low diffusion coefficient (D = 6.44628·10^−12^ m^2^/s) and higher R^2^ value (0.855) confirm that walnut shell addition leads to a reduced and more uniform moisture uptake, enhancing the hydrolytic stability of the composite.

These results are further supported by the degradation kinetics presented in Figure 9b, where the evolution of log(M(t)/M∞) over log(time) reveals the fastest molecular weight decay for PLA15C, reflecting extensive hydrolytic chain scission. Conversely, the degradation profiles of PLA15W and PLA15E remain relatively flat, indicating slower polymer backbone cleavage and better resistance to hydrolytic degradation.

Overall, these findings demonstrate that the type and nature of organic filler significantly affect the water sorption and hydrolytic degradation behavior of PLA composites. Hydrophilic and porous fillers (e.g., coffee grounds, chitin) promote rapid water diffusion and accelerated degradation, while more hydrophobic or compact fillers (e.g., walnut shells) act as effective barriers, improving the long-term dimensional and chemical stability of the biocomposites. These insights are critical for the design of PLA-based materials intended for humid or aqueous environments.

### 3.4. Statistical Analysis

The mechanical performance of PLA-based biocomposites modified with natural fillers exhibited notable variability, as reflected in the standard deviations reported for tensile and flexural properties (Table 7 and Table 8). To systematically assess the dispersion of results and the reliability of observed trends, statistical analyses were conducted. To normalize variability relative to mean values, the coefficient of variation (*CV*) was calculated by Equation (5):

(5) $$CV\;(\%)=(\frac{\pi standard\;deviation}{mean})\times \;100$$

Higher *CV* values were particularly evident in PLA15E and PLA15W, indicating a greater dispersion in tensile performance. This is likely due to poor filler–matrix connection, variable particle morphology, and inconsistent dispersion during processing. In contrast, PLA15S and PLA15C demonstrated low *CVs* (below 2%), suggesting better structural uniformity and reliable interfacial load transfer mechanisms.

The highest variability was observed in PLA15E and PLA15W, particularly in flexural strength (*CV* > 6%), likely due to filler agglomeration, inconsistent interfacial bonding, and morphological irregularities. Conversely, PLA and PLA15C demonstrated low *CVs*, indicating more homogeneous dispersion and reproducible mechanical behavior.

Low *CV* values indicate good filler dispersion quality and structural integrity of the composite, while high *CVs* may indicate processing problems or lack of interfacial compatibility. In particular, PLA15C and PLA15S composites showed the highest repeatability of results, making them more predictable candidates for structural applications.

## 4. Conclusions

This study demonstrated that biocomposites based on PLA and agri-food-derived fillers can be engineered with tunable mechanical and degradation properties. Key findings include the following:

Mechanical performance: All natural fillers improved tensile strength compared to neat PLA, with the seashell (PLA15S) and coffee ground (PLA15C) composites showing the highest enhancements (~55 MPa and ~54 MPa, respectively). However, this reinforcement often came at the cost of reduced impact resistance and flexural strength, especially for PLA15C.

Morphological insights: SEM analysis revealed that filler shape, dispersion, and interfacial compatibility substantially influenced crack propagation and filler retention. Coffee grounds and walnut shells offered better anchoring due to their irregular, lignocellulosic morphology.

Aging behavior: PLA15C showed the greatest hydrolytic stability, with only a 63% reduction in tensile strength after 28 days of water immersion at elevated temperature, significantly outperforming other formulations, especially PLA15W and PLA15E, which suffered nearly complete mechanical degradation.

Structure–property interplay: Fillers with higher calcium carbonate content (e.g., seashell and eggshell powders) contributed to improved initial strength and stiffness, likely due to their rigidity particle dispersion in the matrix. However, they also promoted increased brittleness and reduced resistance to hydrolytic aging. In contrast, lignocellulosic fillers (e.g., spent coffee grounds) did not significantly improve mechanical strength, but their fibrous morphology may have contributed to better mechanical stability over time. Despite their inherent hydrophilicity, the degradation behavior of all composites remained accelerated, suggesting that water uptake and interfacial instability played a dominant role regardless of filler type.

These results validate the potential of post-consumer food waste as a functional filler in biodegradable polymer systems. Future work should focus on improving filler–matrix compatibility via surface functionalization to further enhance durability and expand the application potential of such sustainable composites.

## Figures and Tables

**Table 1:** Basic properties of PLI 005.

Properties	Value	Standard
Mold shrinkage	0.3–0.4%	-
Melting temperature	170–180 °C	-
Max. tensile strength	65 MPa	ISO 527 [40]
Young’s modulus	3.5 GPa	ISO 527 [40]
Impact strength	25 kJ/m^2^	Charpy’s methods ISO 179 [41]

**Table 2:** Fiber loadings (wt%) of different composite specimens.

Sample	PLA (wt.%)	Compatibilizer (wt%)	Seashells (wt%)	Eggs Shells (wt%)	Walnuts Shells (wt%)	Coffee Grounds (wt%)
PLA	97	3	0	0	0	0
PLA15S	82	3	15	0	0	0
PLA15E	82	3	0	15	0	0
PLA15W	82	3	0	0	15	0
PLA15C	82	3	0	0	0	15

**Table 3:** Tensile strength and Young’s modulus of PLA and its composites.

Composites	Tensile Strength [MPa]	Young’s Modulus [MPa]
PLA	53.0 ± 5.3	4415 ± 243
PLA15S	42.3 ± 1.5	5316 ± 80
PLA15E	42.9 ± 3.2	4917 ± 304
PLA15W	30.3 ± 3.4	5030 ± 330
PLA15C	42.9 ± 2.1	530 ± 8

**Table 4:** Flexural strength, flexural modulus, and impact strength of PLA and its composites.

Composites	Flexural Strength [MPa]	Flexural Modulus [MPa]	Impact Strength [kJ/m^2^]
PLA	108 ± 0.7	3473 ± 30	17.94 ± 0.71
PLA15S	92.6 ± 0.35	3742 ± 68	7.79 ± 0.35
PLA15E	89.3 ± 5.75	3939 ± 219	8.75 ± 0.40
PLA15W	85.4 ± 5.95	3641 ± 206	10.65 ± 0.51
PLA15C	59.3 ± 0.35	4330 ± 53	6.08 ± 0.27

**Table 5:** Deformation for tensile and flexural tests before and after aging.

Composites	Deformation [mm]			
	Tensile Test		Flexural Test	
	Before	After	Before	After
PLA	0.35 ± 0.03	0.026 ± 0.0011	6.2 ± 0.11	0.3 ± 0.02
PLA15S	0.29 ± 0.04	0.134 ± 0.0052	5.1 ± 0.09	1.6 ± 0.07
PLA15E	0.24 ± 0.02	0.018 ± 0.0005	4.9 ± 0.21	0.6 ± 0.01
PLA15W	0.17 ± 0.02	0.005 ± 0.000	5.6 ± 0.52	0.6 ± 0.01
PLA15C	0.32 ± 0.03	0.176 ± 0.007	2.7 ± 0.16	1.9 ± 0.08

**Table 6:** Kinetic parameters of hydrolytic degradation of neat PLA and its composites.

Composites	n	log(k)	k	R^2^	D [m^2^/s]
PLA	0.2437	−0.3346	0.463	0.96	7.1824 × 10^−12^
PLA15S	0.2128	−0.279	0.526	0.933	9.0903 × 10^−12^
PLA15E	0.2489	−0.3257	0.472	0.93	7.1824 × 10^−12^
PLA15W	0.2542	−0.3354	0.462	0.855	6.44628 × 10^−12^
PLA15C	0.2896	−0.4822	0.329	0.492	2.51808 × 10^−12^

**Table 7:** Coefficient variation in tensile strength and Young’s modulus.

Composites	Tensile Strength *CV* [%]	Young’s Modulus *CV* [%]
PLA	1.56	1.92
PLA15S	1.82	3.49
PLA15E	3.33	5.68
PLA15W	4.08	4.76
PLA15C	1.30	5.50

**Table 8:** Coefficient variation in flexural strength, flexural modulus and impact strength.

Composites	Flexural Strength CV [%]	Flexural Modulus *CV* [%]	Impact Strength *CV* [%]
PLA	0.65	0.86	3.96
PLA15S	0.38	1.82	4.49
PLA15E	6.44	5.56	4.57
PLA15W	6.97	5.66	4.79
PLA15C	0.59	1.22	4.44

## Data Availability

The original contributions presented in this study are included in the article/supplementary material. Further inquiries can be directed to the corresponding author(s).

## Supplementary Materials

The following supporting information can be downloaded at: https://www.mdpi.com/article/10.3390/ma18153608/s1, Figure S1: Tensile curves of PLA and its composites (a) before and (b) after aging; Figure S2: Flexural curves of PLA and its composites (a) before and (b) after aging.
