# Supplementary material for: Development and Mechanical Characterization of Environmentally Friendly PLA/Crop Waste Green Composites
Source: Materials (Basel). 2025 Jul 31;18(15):3608. doi: 10.3390/ma18153608 (PMC12348436; doi:10.3390/ma18153608)
Supplement: Supplementary file 1 [file materials-18-03608-s001.zip › materials-3732911-supplementary.pdf]

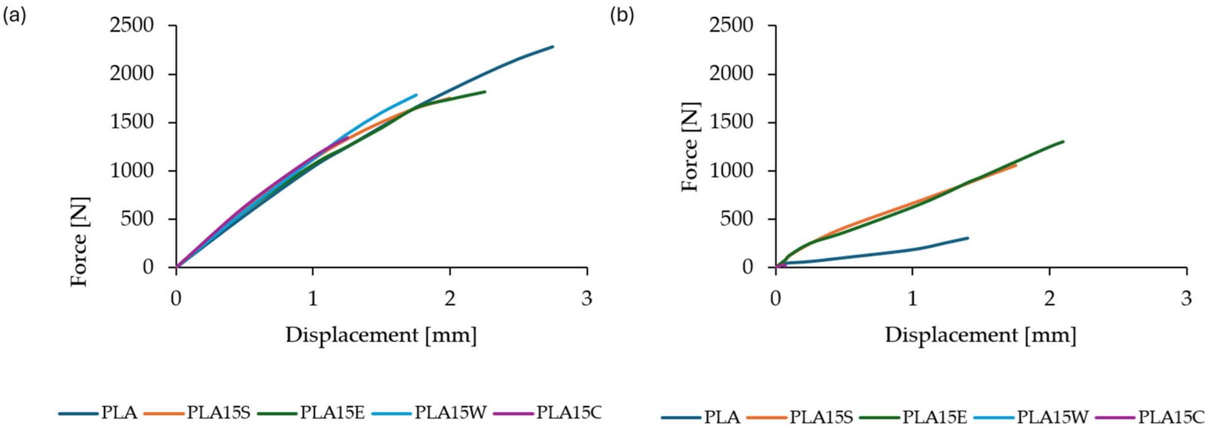

S1. Tensile curves of PLA and its composites (a) before and (b) after aging

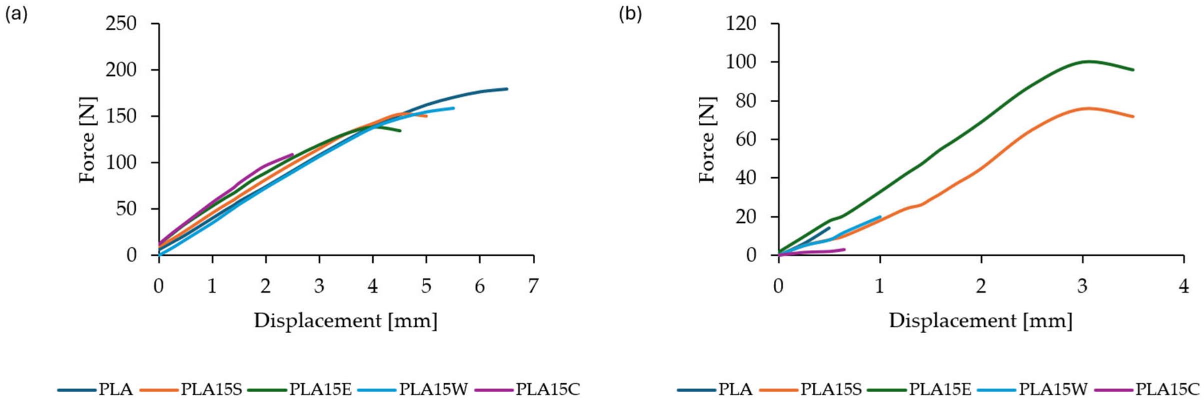

S2. Flexural curves of PLA and its composites (a) before and (b) after aging
